# Population genetics of *Anopheles funestus*, the African malaria vector, Kenya

**DOI:** 10.1186/s13071-018-3252-3

**Published:** 2019-01-08

**Authors:** Edwin O. Ogola, Joel O. Odero, Joseph M. Mwangangi, Daniel K. Masiga, David P. Tchouassi

**Affiliations:** 1 International Centre of Insect Physiology and Ecology (icipe), P.O. Box 30772-00100, Nairobi, Kenya; 2 Centre for Geographic Medicine Research Coast, Kenya Medical Research Institute (KEMRI), P.O. Box 42880-108, Kilifi, Kenya

**Keywords:** *Anopheles funestus*, Malaria vector, Microsatellites, Kenya, Malaria risk zones, Population genetics

## Abstract

**Background:**

*Anopheles funestus* is among the major malaria vectors in Kenya and sub-Saharan Africa and has been recently implicated in persistent malaria transmission. However, its ecology and genetic diversity remain poorly understood in Kenya.

**Methods:**

Using 16 microsatellite loci, we examined the genetic structure of *An. funestus* sampled from 11 locations (*n* = 426 individuals) across a wide geographical range in Kenya spanning coastal, western and Rift Valley areas.

**Results:**

Kenyan *An. funestus* resolved as three genetically distinct clusters. The largest cluster (FUN1) broadly included samples from western and Rift Valley areas of Kenya with two clusters identified from coastal Kenya (FUN2 and FUN3), not previously reported. Geographical distance had no effect on population differentiation of *An. funestus*. We found a significant variation in the mean *Plasmodium* infectivity between the clusters (*χ*^2^ = 12.1, *df* = 2, *P* = 0.002) and proportional to the malaria prevalence in the different risk zones of Kenya. Notably, there was variation in estimated effective population sizes between the clusters, suggesting possible differential impact of anti-vector interventions in represented areas.

**Conclusions:**

Heterogeneity among Kenyan populations of *An. funestus* will impact malaria vector control with practical implications for the development of gene-drive technologies. The difference in *Plasmodium* infectivity and effective population size between the clusters could suggest potential variation in phenotypic characteristics relating to competence or insecticide resistance. This is worth examining in future studies.

**Electronic supplementary material:**

The online version of this article (10.1186/s13071-018-3252-3) contains supplementary material, which is available to authorized users.

## Background

The burden of malaria remains high in Africa despite the gradual decline that has been witnessed over the last decade. As of 2016, Africa accounted for > 90% of the 445,000 malaria deaths and 216 million cases were recorded worldwide [1]. The burden is, however, not uniform and characterized by spatio-temporal variability in different parts of Africa [2, 3]. In Kenya, the disease has been on the increase in some parts but with stable or declining parasite infection rates in other areas [2, 4]. Heterogeneity within vector populations could impact on spatio-temporal trends in malaria parasite transmission [5, 6]. This underscores the need to characterize the genetic structure of key vectors at a national scale for better assessment of the impacts on malaria control efforts [6, 7].

*Anopheles funesus* (*s.s.*) (hereinafter referred to as *Anopheles funestus*) is one of the four major malaria vector species widely distributed throughout tropical Africa and a key vector in Kenya [8, 9]. It is the nominal species and primary vector in the *An. funestus* group which comprises at least 13 sibling species [8]. The species is highly susceptible to malaria parasites and has a strong preference to feed on humans which endows it with a high vectorial capacity [10, 11]. Its potential capacity for rapid evolutionary adaptation is seen in the exhibition of divergent traits in response to scale up of long-lasting insecticidal net (LLIN) distribution. These include a shift toward diurnal [12] and outdoor feeding [13] habits, and development of multiple insecticide resistance mechanisms [14]. These biological and phenotypic traits relevant to disease epidemiology can be genetically determined with potential influence on the evolution of vector species or populations [15].

There have been a few studies on the population structure of *An. funestus* in Kenya. These date back to more than a decade ago preceding large scale LLIN measures which are known to have influenced vectorial systems and potentially associated selective adaptive responses in malaria vectors [16, 17]. Based on chromosomal inversions, Kamau et al. [18] observed levels of genetic differentiation among Kenyan *An. funestus* populations. A few studies in Kenya have employed microsatellite markers in determining the genetic structure of *An. funestus*, known to be highly informative for fine-scale population genetic analysis and lineage reconstruction [19, 20]. Braginets et al. [21] using microsatellites, found differentiation between *An. funestus* populations from coastal and western Kenya. This study was, however, limited in scope with just four sample sites (two from each region) and five loci. A pan-African microsatellite-based study found two population subdivisions in *An. funestus* [22]; however, this was not so useful for national scale inference. Delineating the fine-scale population structure of disease vectors such as *An. funestus* is crucial for understanding their epidemiological significance and their potential response to current and future vector control measures [6].

Effective malaria control towards elimination targeting vectors in Kenya will benefit from improved knowledge of the genetic heterogeneities of vector populations, especially *An. funestus*. Being among the key malaria vectors in Africa, this species has recently been implicated in persistent malaria transmission [23] with increases in the relative abundance following roll-out of LLINs in parts of Kenya [4]. Furthermore, the success of gene drives as developing strategies in the fight against malaria [24, 25] hinges on knowledge of the extent of genetic relatedness among local population of the target species. As part of the HEG Target Malaria Project, this study used 16 microsatellite loci to investigate the genetic structure of geographically distinct *An. funestus* samples from 11 locations spanning diverse malaria endemicities (coastal, western, Rift Valley) of Kenya. Our goal was to quantify the population structure of *An. funestus* with the objective of associating identified genotypes with geographical locations and explore possible links with malaria endemicity based on *Plasmodium* infection prevalence.

## Methods

### Sampling

**Fig. 1:**
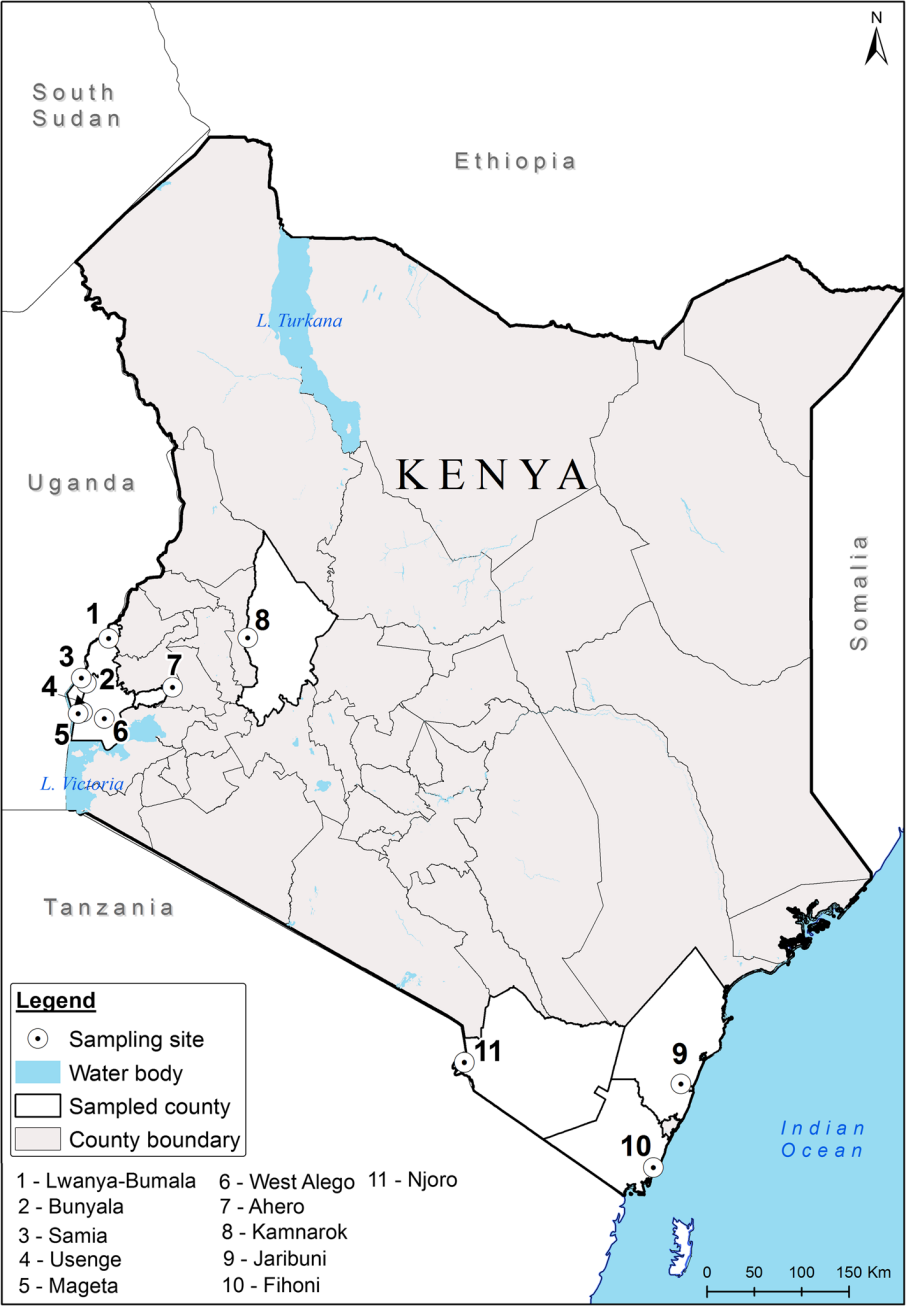
Map of Kenya showing the sampling locations

We used DNA of *An. funestus* (*s.s.*) (hereinafter referred to as *An. funestus*) samples reported in a previous study [9]. The samples were from 11 sites spanning malaria risk areas in coastal, Rift Valley and western Kenya (Fig. 1, Table 1). The samples were identified using primers targeting the internal transcribed spacer region 2 (ITS2) of ribosomal DNA [26] as previously described [9].

**Table 1:** *Anopheles funestus* sampling sites in Kenya and number of specimens analyzed

Region	County	Location (abbreviation)	Malaria endemicity^a^	Collection date (DD/MM/YY)	Collection method	Sample size
Western	Kisumu	Ahero (H)	Lake Endemic	4–16/11/2015	CDC (Out)	68
	Siaya	Usenge (US)	Lake Endemic	23/7/2015	IR	31
	Siaya	West Alego (AL)	Lake Endemic	1/7/2017	CDC (In/Out)	61
	Siaya	Mageta (MAG)	Lake Endemic	8/6/2014	IR	8
	Busia	Bunyala (BUN)	Lake Endemic	3/7/2017	CDC (In/Out)	51
	Busia	Samia (SAM)	Lake Endemic	30/6/2017	CDC (In/Out)	102
	Busia	Lwanya-Bumala (LN)	Lake Endemic	3/7/2017	CDC (In/Out)	17
Rift Valley	Baringo	Kamnarok (B)	Highland Epidemic and semi-arid, seasonal	12/8/2015	IR	11
Coastal	Kilifi	Jaribuni (J)	Coast Endemic	8/6/2017	CDC (In/Out)	31
	Kwale	Fihoni (FH)	Coast Endemic	7/6/2017	CDC (In/Out)	22
	Taita-Taveta	Njoro (T)	Coast Endemic	6/7/2017	IR	24

Collection methods included indoor resting (IR) and CDC light traps (CDC) conducted indoors (In) and/or outdoors (Out)

^a^[2]

### Detection of *Plasmodium* malaria parasites

We further screened the specimens for *Plasmodium* infections using PCR and melting analysis of amplicons in a RotorGene Q thermocycler (Qiagen, Hilden, Germany) targeting non-coding mitochondrial sequences (ncMS) [27] and/or amplification of the cytochrome c oxidase subunit 1 (*cox*1) gene [28] as previously described [9].

### Microsatellite genotyping

*Anopheles funestus* samples were genotyped at 16 polymorphic microsatellite markers spanning the genome [29, 30]. The markers were optimized into four multiplexes based on suitable primer annealing temperatures and non-overlapping expected allele size ranges (Additional file 1: Table S1) using the program Multiplex Manager v.1.0 [31]. Microsatellite detection from gDNA samples from each site (8–102 per site, *n* = 426) were run using the Type-it Microsatellite PCR kit (Qiagen): 6.25 μl of the 2X Type-IT master Mix, 2.5 μl of primer mix (2 μM each), 1 μl of RNase-free H_2_O and 4 μl of gDNA as template (10 ng/μl ≤ x ≤ 20 ng/μl). Thermal cycling conditions in a SimpliAmp Thermal Cycler (Applied Biosystems, Loughborough, UK) were as follows: initial denaturation at 95 °C for 5 min; followed by 35 cycles of denaturation at 95 °C for 30 s, annealing (at 52–57 °C, depending on the multiplex) for 90 s and extension at 72 °C for 30 s; with a final extension at 60 °C for 30 min. Next, 1.25 μl of the PCR product was reconstituted in 3.75 μl of water and outsourced for fragment analysis at the DNA Sequencing Facility, University of Illinois at Urbana-Champaign, Urbana, Illinois, USA. The PCR fragments were separated on an ABI 3730XL (Applied Biosystems) sequence analyzer using the GeneScan™ 500LIZ™ size standard. The allele sizes were scored using GeneMarker software v.2.6.7 (SoftGenetics, LLC, Pennsylvania, USA) with each allele size score double-checked manually. Genotyping errors possibly due to null alleles, large allele dropouts and scoring of stutter peaks were initially checked using MICRO-CHECKER v.2.2.3 [32].

We estimated genetic diversity in the different geographical populations by calculating number of alleles per locus per population (*A*), allelic richness (*R*_*S*_), estimated population differentiation (*F*_*ST*_) and inbreeding coefficients (*F*_*IS*_) per locus for each pair of population using FSTAT v.2.9.3 [33]. We assessed linkage disequilibrium (*LD*) between locus pairs within each population and deviations from Hardy-Weinberg equilibrium tested using Markov chain default parameters in GENEPOP [34]. An analysis of molecular variance (AMOVA) was performed on all geographical populations and a principle coordinates analysis (PCoA) based on *F*_*ST*_ run in GenAIEx v.6.5 [35]. We further plotted a neighbor-joining tree using pairwise population *F*_*ST*_ estimates to compare the structure of the different populations. Population structure was analyzed using Bayesian cluster analysis software STRUCTURE v.2.3.4 [36] using the admixture model with 50,000 for burn-in, 100,000 iterations and repeated 20 times for each value of *K* ranging from 1 to 12. The most likely number of populations, *K*_*MAX*_, was estimated using the Evanno method [37] implemented in CLUMPAK [38]. A correlation analysis comparing the genetic distance and geographical distance for all populations was conducted using a Mantel test with 100,000 randomizations in IBD v.1.52 [39]. To investigate whether any of the populations experienced recent genetic bottlenecks, a Wilcoxon sign-rank test for heterozygosity excess was applied under a two-phase model (TPM) with 20% non-single step mutation using the program Bottleneck v.1.2.02 [40].

## Results

### Genetic diversity in *Anopheles funestus* populations

All samples amplified reliably across the 16 loci showing the robustness of the multiplex design. The number of samples in each population ranged between 8–102 individuals with 8 of the 11 populations having ≥ 20 individuals (Table 1).

Genetic diversity estimates across all populations are shown in Additional file 1: Table S2. The levels of microsatellite polymorphism across loci and samples were moderate to high with the mean observed heterozygosity (*H*_*O*_) values within each of the geographical mosquito populations ranging from 0.48 in Njoro to 0.74 in Lwanya-Bumala. Following adjustment for variances in the number of individuals in each population, allelic richness (*A*_*R*_) ranged from 3.3 in Njoro to 5.1 in Ahero. We made a total of 1320 pairwise comparisons for *LD* of which 84 (7%) were significant (*P* < 0.005). Analysis of molecular variation (AMOVA) indicated significant molecular variation within and among the populations with 84% of the variation within individuals, 10% among individuals, and 6% among populations. The pairwise estimates of genetic differentiation among the 11 geographical populations ranged from -0.002 between Ahero and Lwanya -Bunyala to 0.32 between Njoro and Jaribuni (Table 2).

**Table 2:** Pairwise comparison of genetic diversity (*F*_*ST*_) among the 11 geographical *A. funestus* populations sampled

	Ahero	Kamnarok	Usenge	Mageta	Bunyala	West Alego	Njoro	Jaribuni	Lwanya-Bumala	Fihoni
Kamnarok	0.0184									
Usenge	0.0047	0.0252								
Mageta	0.0161	0.0336	**0.0086**							
Bunyala	-0.0017	0.0341	0.0043	**0.0122**						
West Alego	0.0028	0.0329	0.0065	**0.0209**	0.0002					
Njoro	0.2553	0.2995	0.2798	0.2957	0.2734	0.2554				
Jaribuni	0.092	0.0924	0.0949	0.1125	0.0896	0.0866	0.3235			
Lwanya-Bumala	0.0137	0.017	0.0196	0.0322	0.0149	0.0132	0.2814	0.0889		
Fihoni	0.0482	0.0618	0.0526	0.0782	0.0465	0.0353	0.3132	0.0389	0.0491	
Samia	0.01	0.038	0.0117	**0.0285**	0.0089	0.0066	0.2665	0.1005	0.0256	0.0353

The bold values indicate comparisons that were significant following Bonferroni correction

**Fig. 2:**
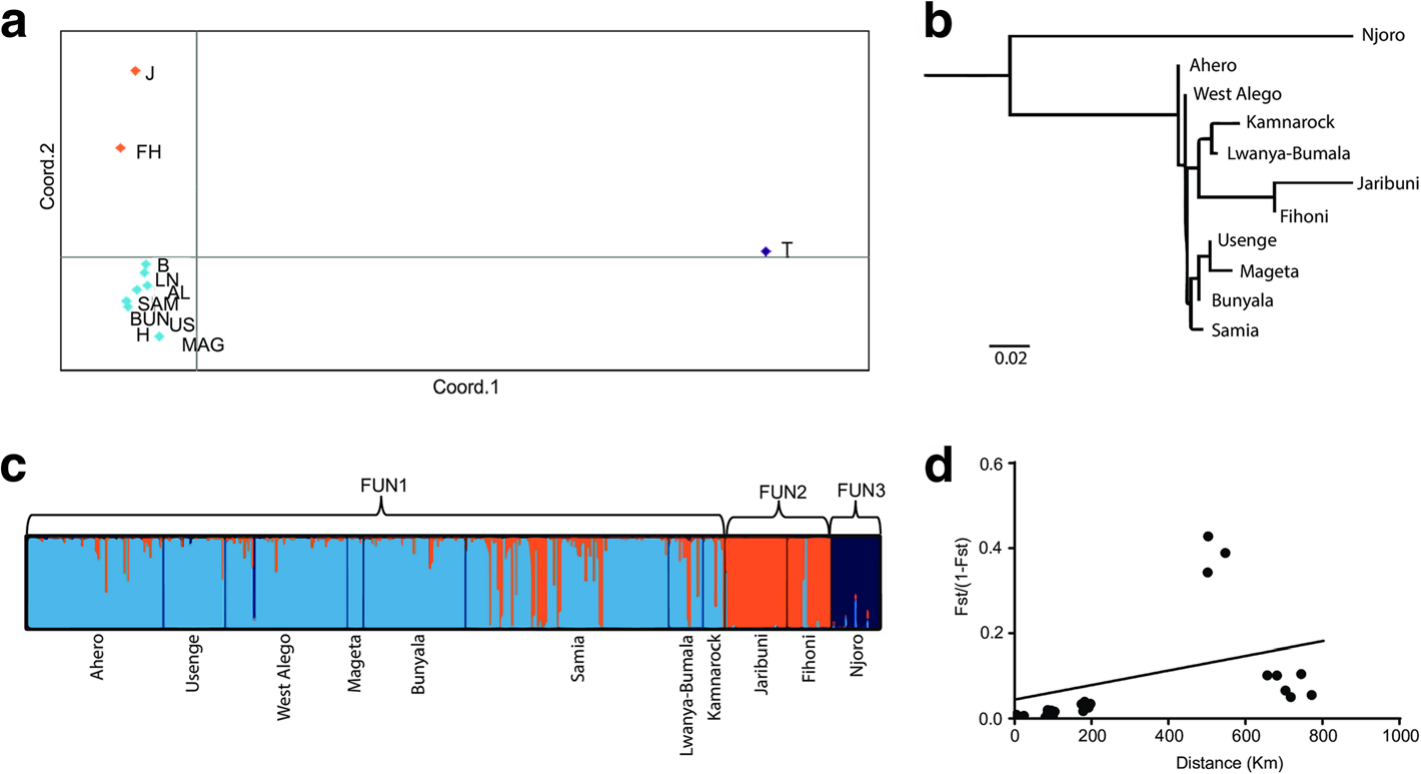
Population structure of *Anopheles funestus* in Kenya. **a** Principle coordinates analysis plot based on pairwise population *F*_*ST*_ estimates showing three clusters. **b** Neighbor-joining tree based on the *F*_*ST*_ pairwise estimates using 16 microsatellites. **c** Bayesian-based structure patterns K = 3 showing separation into 3 distinct clusters. **d** Isolation by distance comparing genetic distance *versus* geographical distance based on the Mantel test. The cluster FUN1 broadly include sites from western and Rift Valley, Kenya; coastal clusters FUN2 (Fihoni, Jribuni) and FUN3 (Njoro-Taita Taveta). Site abbreviations are indicated as in Table 1

To visualize the genetic relatedness among individuals, we first performed PCoA. The top three PCoA components explained 54.21, 18.25 and 9.95% of the total variance and grouped the individuals into three main clusters (Fig. 2a). Similarly, a neighbor-joining (NJ) tree, based on pairwise *F*_*ST*_ estimates, showed three distinct populations for *An. funestus* (Fig. 2b). In support of population structuring, Bayesian clustering analysis with STRUCTURE showed that three population clusters (k = 3) best explain the genetic variance present in our data (Fig. 2c, Additional file 2: Figure S1). The first and largest subgroup (hereafter FUN1) comprised most *An. funestus* specimens collected in western Kenya (Ahero, Usenge, Lwanya-Bumala, Bunyala, W. Alego, Samia and Mageta Island) and Kamnarok, a riverine area of Baringo County in the Rift Valley. The second group (hereafter FUN2) included individuals from the coastal Kenyan sites of Fihoni and Jaribuni, while the smallest subgroup (hereafter FUN3) was associated with individuals exclusive to Njoro (Taita Taveta County) (Fig. 2c). The membership coefficients of geographical samples to their respective clusters/subgroup were relatively high, ranging between 79–98% for subgroup FUN1, 92–97% for subgroup FUN2 and 96% for subgroup FUN3 (Table 3). We further examined the extent of differentiation between the three subgroups within *An. funestus* by estimating the genotypic differentiation in GENEPOP using Markov chain default parameters. We found strong divergence among the clusters (*P* < 0.005) corroborating patterns of population structure observed within the species. Mantel tests on correlation between pairwise genetic and geographical distances indicated a weak and non-significant positive association between genetic and geographical distances (Mantel test: *r* = 0.33, *P* = 0.798) (Fig. 2d).

**Table 3:** Proportion of memberships in each of the three identified clusters

Population	Group	Cluster			
		1 (%)	2 (%)	3 (%)	*n*
Ahero	FUN1	95	5	1	68
Kamnarok	FUN1	81	18	0	11
Usenge	FUN1	96	3	1	31
Mageta	FUN1	98	1	1	8
Bunyala	FUN1	96	3	0	51
West Alego	FUN1	94	4	2	61
Samia	FUN1	79	20	0	102
Lwanya-Bumala	FUN1	79	20	1	17
Jaribuni	FUN2	3	97	0	31
Fihoni	FUN2	8	92	0	22
Njoro	FUN3	3	1	96	24

### Phenotypic patterns across identified clusters

Having observed three distinct clusters, we asked whether there is any phenotypic variation among these populations by examining the *Plasmodium* infectivity as a measure of malaria risk. We thus assessed the proportion of *Plasmodium* infectivity attributable to *An. funestus* of the total *An. funestus* group specimens analyzed per site representative of each cluster. Results revealed that mean *Plasmodium* sporozoite infectivity varied significantly between the clusters (*χ*^2^ = 12.1, *df* = 2, *P* = 0.002) being 8.4% (52/619; 95% CI: 6.4–10.9%), 3.1% (10/324; 95% CI: 1.6–5.8%**)** and 0% (0/29; 95% CI: 0–14.6%**)** for clusters FUN1, FUN2 and FUN3, respectively. We next estimated the effective populations (Ne) for each cluster using NeEstimator v.2 (Do et al. [41] based on linkage disequilibrium (*LD*). We found Ne to be 483.9 (95% CI: 419.8–566.9), 17.7 (95% CI: 15.0–20.9) and 71.2 (95% CI: 30.9–∞) for FUN1, FUN2 and FUN3, respectively.

## Discussion

Gillies & De Meillon [42] had noted several polymorphic inversions in *An. funestus* and suggested possible genetic differentiation into locally adapted populations throughout its distribution range in tropical Africa. Collins & Besansky [10] further posited that greater discontinuities are not unexpected for this species given its breeding habitat in semi-permanent water bodies. The population genetic structure of this species has, however, been found to be shallow within local scales [22]. A similar shallow resolution has been observed for this species in West Africa using a combination of markers (chromosomal inversions, microsatellites and mitochondrial *nad*5 gene), with notable microsatellite differentiation of chromosomal forms facilitated by chromosome 3R inversions [43]. Here, we found *An. funestus* to be genetically subdivided across its range in Kenya. Most of the genetic variation was accounted for by within-population differences among individuals, consistent with previous findings [22, 43]. Nonetheless, we found population subdivision resulting in three genetically distinct clusters within *An. funestus* supported by Bayesian clustering (structure) analysis, NJ phylogeny and PCoA (Fig. 2a-c)*.* The largest cluster (FUN1) broadly included samples from western Kenya (Ahero, Usenge, Bunyala, West Alego, Lwanya-Bumala, Samia, Mageta) and Kamnarok in the Rift Valley. Two clusters were recovered from coastal Kenya: FUN2 comprising samples from Jaribuni (Kilifi County) and Fihoni (Kwale County) and FUN3 unique to Njoro (Taita-Taveta County). Our findings indicate a much higher genetic diversity and subdivision for this species than previously reported in Kenya [18, 21]. Previous studies have highlighted significant genetic population differentiation between coastal and western Kenya populations, although with no evidence for genetic structure within coastal populations. Our recovery of additional structure in coastal Kenya could be attributed to the sampling scale with more sites and high number of loci employed.

Improving our understanding of the population structure and what drives genetic differentiation among mosquitoes will inform effective disease control. Heterogeneity within vector species resulting from evolution contributes to variability in malaria cases spatially and temporally [5, 6]. A few studies have examined patterns of genetic variation in relation to malaria epidemiological outcomes [44]. We found representation of samples within the clusters mirroring the degree of malaria endemicity or prevalence in Kenya. Western Kenya (represented by cluster FUN1) classified as Lake Endemic risk zone remains the hotbed of malaria in Kenya with a recent overall malaria prevalence of 27% [2]. About three-fold lower prevalence was estimated for the coastal region averaging 8%. Estimated mean *Plasmodium* infectivity attributed to *An. funestus* was 8.4% (52/619) for FUN1 (western Kenya) and 3.1% (10/324) for the coastal cluster FUN2, proportional to the mean malaria prevalence in the different risk zones of Kenya as highlighted earlier. Specimens from Kamnarok (riverine area in the Rift Valley) strongly assigned to the western cluster FUN1 (Fig. 2c). This site is a malaria hotspot within Baringo County with the overall highest prevalence rate of 23%, compared to less than 1% for the rest of the county [45]. Furthermore, estimated effective population sizes for *An. funestus* were 6 to 28-fold higher in cluster FUN1 (483) representing western Kenya, compared with the clusters from coastal Kenya [17 (FUN2) and 71 (FUN3)], a trend also exhibited in the genetic diversity measured by the number of alleles and observed heterozygosity (Additional file 1: Table S2). The size of a vector population correlates directly with its vectorial capacity [46]. Our findings on estimated population sizes are consistent with field data that indicate an increase in abundance of *An. funestus*, especially in western Kenya in recent times [4] following the dwindling importance *Anopheles gambiae* (*s.s.*) in malaria transmission. Given that efficient transmission translates to more infectivity in mosquitoes [47], our findings suggest variation in vectoral capacity among discrete populations of *An. funestus* which may impact differently on the epidemiology of malaria transmission across the different risk zones of Kenya. We recognize, however, that variation in mosquito infectivity rates could vary between sites [9, 48] likely influenced by other processes. For instance, different malaria transmission settings will impose a different malaria “reservoir” size in the human population, contributing to differences in mosquito infection rates. Nonetheless, it is worth noting that not all infections translate into infectivity, likely determined in part by vector factors (e.g. age structure, vector competence, biting preferences) [49] which may vary among the different clusters found in this study. Such difference in effective population size between the clusters could also relate to the extent anti-vector interventions in the represented areas as previously reported for other malaria vectors in West Africa [46]. Future research examining correlation in genetic structuring and malaria transmission should consider the contribution of other possible anopheline vectors within a given locality.

The Rift Valley has been identified as a barrier to gene flow in *An. funestus* [18] and *An. gambiae* [50], although the extent so far remains clear. While *An. funestus* has been documented in the Rift Valley [9], we found specimens from the riverine area (Kamnarok) of Rift Valley were strongly assigned to the western cluster, FUN1 (Fig. 2c). Further studies including samples from varied sites covering either side of the Rift Valley will shed light on the role of the Rift Valley in genetic structuring of this species. Our data found a weak positive correlation between geographical and genetic distance in *An. funestus*, indicative of strong genetic differences between clusters and possibly local adaptation. Samples from sites separated by between 5–97 km in western Kenya grouped together in cluster FUN1. We did not find any evidence of population bottlenecks among any of the population clusters.

The underlying cause for the population divergence observed between *An. funestus* in coastal and western Kenya remains unclear. We mainly encountered *An. funestus* indoors in western Kenya (FUN1) and outdoors at the coast (FUN2) (data not shown). Could the genetic and behavioral divergence be related to differences in the scale of vector control interventions between the regions or an effect of climate change? Such anti-vector interventions have been found also to impact population size of vector populations [46]. Mass distribution of long-lasting insecticidal bednets (LLINs) have taken place in Kenya since 2006 [51]. Insecticide resistance in vector populations has been widespread with large scale exposure resulting in altered abundance, behavioral shifts and general ecology of major vector populations (e.g. *An. funestus*, *An. gambiae*) [16]. Changes in the distribution among mosquitoes in the *An. funestus* group is evident where *An. funestus* remains the dominant sibling species in western Kenya and *An. rivulorum* in coastal Kenya, represented by cluster FUN2 [9]. As noted in parts of western Kenya, *Anopheles funestus* has reemerged as main malaria vector despite widespread use of insecticide-treated bednets, partly attributed to insecticide resistance [52]. Recently, genomic signatures of a major recent population decline of *An. gambiae* in coastal Kenya was reported, although not attributed to ITNs usage [53]. On the other hand, there are records of *An. funestus* having been eliminated from parts of Africa due to prolonged severe drought, e.g. [54], suggesting extreme climate variability can affect the survival of this species. Such a negative relationship between prolong drought and *An. funestus* occurrence raises the possibility of extreme climatic patterns in influencing the structuring among species. Western Kenya receives more rainfall than coastal Kenya and a combination of possible differences in the scale of interventions and climatic factors may be impacting on *An. funestus* population dynamics. Certainly, the factors driving the population structure of *An. funestus* in Kenya deserve further research.

Malaria vector control in Kenya will benefit from improved knowledge of the genetic heterogeneities within populations of *An. funestus* and their effects on malaria transmission. The persistent high transmission attributed to this species in western Kenya and mainly encountered indoors [9] will inform targeted measures such as indoor residual sprays (IRS). Pronounced genetic structure uncovered for this major malaria vector in Kenya has practical implications for the implementation of gene-drive technologies for mosquito control. However, extensive sampling of multiple populations will be needed to reveal the extent of the variation; this will help inform the design of such an approach, robust to natural genetic variation. Perhaps such a genetic approach will require multiple release points and may be more promising in western Kenya given the relative genetic uniformity. Since holistic malaria control using gene drive approaches needs to target multiple major malaria vectors [25], similar genetic studies should be extended to other vectors like *Anopheles arabiensis* which occurs in sympatry with *An. funestus* in most environments of Kenya and Africa as a whole.

## Conclusions

We have unraveled subdivisions within *An. funestus* in Kenya revealing three genetically distinct clusters. We found variation in mean *Plasmodium* infectivity between the *An. funestus* clusters proportional to the mean malaria prevalence across risk areas of Kenya. This association does not, however, prove causality, as other processes could have contributed to the observed result. A holistic examination of all anophelines contributing to transmission in a given focus and their evolutionary pattern will shed light on the link between transmission and human malaria prevalence. Vector surveillance is integral to malaria elimination efforts, given vectors’ remarkable capacity for evolution and the need for fine-tuning control strategies in the event of changes in local transmission [55]. Most importantly, it is essential to start collecting population genomic data prospectively as an integral part of vector control interventions, to identify their responses to such measures, or the underlying cause of genetic structure and high population size of this species in western compared to coastal Kenya as observed in our data. As pointed out by the *Anopheles gambiae* Genomes Consortium [53], each intervention needs to be treated as an experiment and its effect analyzed on both mosquito and parasite populations. Only then can we improve the efficacy and sustainability of future interventions, while at the same time learn about basic processes in ecology and evolution.

## Additional files

Additional file 1: **Table S1.** Multiplex design and primer details of the 16 microsatellite markers used to study the genetic population structure of *An. funestus* in Kenya. **Table S2.** Genetic diversity across all populations. (DOCX 37 kb)

Additional file 2: **Figure S1.** Evanno delta *K*, STRUCTURE results for *K* = 3 based on microsatellite clustering analysis. (TIF 118 kb)

## Abbreviations

CDC: Centers for Disease Control and Prevention
*cox*1: Cytochrome *c* oxidase subunit 1
IR: Indoor resting
LLIN: long-lasting insecticidal net
ncMS: Non-coding mitochondrial sequences
PCR: Polymerase chain reaction
*s.l.*: *sensu lato*
*s.s.*: *sensu stricto*
