# Supplementary material for: Population genetics of Anopheles funestus, the African malaria vector, Kenya
Source: Parasit Vectors. 2019 Jan 8;12:15. doi: 10.1186/s13071-018-3252-3 (PMC6323828; doi:10.1186/s13071-018-3252-3)
Supplement: Supplementary file 1 — Table S1. Multiplex design and primer details of the 16 microsatellite markers used to study the genetic population structure of An. funestus in Kenya. Table S2. Genetic diversity across all populations. (DOCX 37 kb) [file 13071_2018_3252_MOESM1_ESM.docx]

**Additional file 1: Table S1.** Multiplex design and primer details of the 16 microsatellite markers used to study the genetic population structure of *An. funestus* in Kenya. Initial letters on the sequences indicate the dye used.

| **Plexes** | **Locus** | **Chromosome** | **Sequence (5′ - 3′)** | **Size range** |
| --- | --- | --- | --- | --- |
| **Plex 1** | AFND40 | 2R 9B | **PET**-ATT CAT CCT GTG ATG CTT TG | 188-200 |
|  |  |  | AGG CTC TTC TTT GCA CTG T |  |
|  | FUNL | 2L 24C | **VIC**-AAC AGT GGA AGG CAA ATT GC | 140-166 |
|  |  |  | GCA CGG TTA CCA CTG CTC A |  |
|  | AFND19 | 2L 23A | **6-FAM**-CAG AAC CAC TTC GAT TCA AC | 172-205 |
|  |  |  | CCT GCA CTC AGA AAC ACA C |  |
|  | FUNG | 3R 35A | **NED**-GAG CAA GCA GCT TACT GCA C | 146-168 |
|  |  |  | ACG TTC AGT GCA CA TCAA TG |  |
| **Plex 2** | FUNQ | X 5D | **VIC**-GCA AAC TGC TAG TAA ATG TTT CC | 84-98 |
|  |  |  | ACA TTT CCA CAA TTT GCG C |  |
|  | FUNO | 2R 18A | **NED**-GCA CAC ATT TCA GGC AGC | 110-132 |
|  |  |  | GCC CAC ATT CTG CAC CTT |  |
|  | AFND20 | 3R 32D | **6-FAM**-AGA ACC ACA TTA GGG AAC AG | 123-139 |
|  |  |  | TTT ACA ACC AGC AGC ACA C |  |
|  | AFND30 | 2R 13C | **PET**-GTT CAG CTG TTG GTG TGT TAG | 83-107 |
|  |  |  | TTT TCG TAC GGA GAA AAA TG |  |
| **Plex 3** | AFUB11 | 2L 20D | **6-FAM**-CAG TTT CTG CGT GGA GGA AT | 174-214 |
|  |  |  | AGC AGC TGA TGA GCC ATC TC |  |
|  | AFND7 | 3R 33A | **PET**-TGC ATC ATT CGA CTC GGA AG | 70-84 |
|  |  |  | AAC GGC ACT ACC GTT CAC TG |  |
|  | AFUB6 | 2R 11A | **VIC**-CCA GCA GGT GTG GAG GAC | 134-174 |
|  |  |  | GTC GTA CAA AAG CAC CAC CA |  |
|  | AFND12 | X 3B | **NED**-GTT CTC CAT CGC TGT TCT ACT C | 87-107 |
|  |  |  | TAT AAC GTT TCG TAC ACA CGC C |  |
| **Plex 4** | AFUB12 | 3L 46C | **VIC**-TGG GGA ACT GGT CGT TAG AG | 141-101 |
|  |  |  | CTG GTG ATG GGA TTG AGG AT |  |
|  | FUNF | 3L 43A | **6-FAM**-GCC TTC AGT TTC GAT TGG CG | 104-118 |
|  |  |  | AAT AAG ATG CGA CCG TGG C |  |
|  | AFUB10 | 2L 26CD | **NED**-TGT CCA TGT ACA ACC GCA AC | 178-218 |
|  |  |  | TTC TCC AGC ATC ATC AGC AC |  |
|  | AFND5 | 2R 15C | **PET**-CCT CTC GTT GTG TTG CCT AC | 169-183 |
|  |  |  | GTT CAT ACG TTG CCC GAT TT |  |

**Additional file 1: Table S2.** Genetic diversity across all populations

|  |  | Ahero | Kamnarok | Usenge | Mageta | Bunyala | W. Alego | Njoro | Jaribuni | Lwanya | Fihoni | Samia |
| --- | --- | --- | --- | --- | --- | --- | --- | --- | --- | --- | --- | --- |
| AFND19 | Hex | 0.982 | 0.495 | 0.887 | 0.904 | 0.563 | 0.694 | 0.964 | 0.036 | 0.054 | 0.010 | 0.315 |
|  | Hdef | 0.018 | 0.753 | 0.118 | 0.183 | 0.445 | 0.307 | 0.040 | 0.982 | 0.989 | 1.000 | 0.686 |
|  | *Fis* | 0.087 | 0.040 | 0.033 | 0.273 | 0.103 | -0.028 | 0.254 | -0.250 | -0.200 | -0.314 | -0.045 |
|  | *Ar* | 6.232 | 4.904 | 6.065 | 4.876 | 5.405 | 5.642 | 3.849 | 3.413 | 5.084 | 4.391 | 5.437 |
| AFND40 | Hex | 0.901 | 0.191 | 0.864 | 0.355 | 1.000 | 0.395 | 0.842 | 0.969 | 0.479 | 0.130 | 0.013 |
|  | Hdef | 0.101 | 0.968 | 0.137 | 0.680 | **0.000** | 0.606 | 0.233 | 0.031 | 0.642 | 0.918 | 0.987 |
|  | *Fis* | -0.027 | 0.034 | -0.133 | -0.231 | 0.251 | -0.066 | 0.145 | -0.213 | 0.101 | -0.168 | -0.143 |
|  | *Ar* | 5.472 | 5.702 | 4.888 | 3.889 | 5.140 | 4.488 | 2.745 | 3.861 | 4.666 | 3.954 | 4.166 |
| FUNG | Hex | 0.646 | 0.434 | 0.999 | 1.000 | 0.976 | 0.861 | 0.130 | 0.343 | 0.671 | 0.760 | 1.000 |
|  | Hdef | 0.354 | 0.620 | 0.001 | 0.005 | 0.024 | 0.139 | 0.936 | 0.657 | 0.394 | 0.242 | **0.000** |
|  | *Fis* | 0.039 | 0.072 | 0.459 | 0.800 | 0.222 | 0.012 | -0.268 | -0.170 | 0.018 | -0.176 | 0.034 |
|  | *Ar* | 5.025 | 2.996 | 2.787 | 3.000 | 5.200 | 5.379 | 2.665 | 4.289 | 4.783 | 4.550 | 5.183 |
| FUNL | Hex | 0.706 | 0.981 | 0.982 | 0.995 | 1.000 | 0.976 | 0.998 | 0.654 | 0.691 | 0.247 | 0.973 |
|  | Hdef | 0.294 | 0.019 | 0.019 | 0.016 | **0.000** | 0.025 | 0.002 | 0.346 | 0.398 | 0.851 | 0.027 |
|  | *Fis* | 0.015 | 0.288 | 0.187 | 0.360 | 0.263 | 0.067 | 0.043 | -0.197 | -0.012 | -0.049 | 0.005 |
|  | *Ar* | 7.513 | 5.702 | 6.850 | 6.660 | 7.630 | 7.083 | 3.442 | 5.440 | 5.684 | 5.122 | 5.728 |
| AFND20 | Hex | 0.837 | 0.619 | 0.486 | 0.973 | 0.969 | 0.995 | 0.826 | 0.977 | 0.826 | 0.622 | 0.211 |
|  | Hdef | 0.163 | 0.529 | 0.543 | 0.088 | 0.031 | 0.005 | 0.219 | 0.023 | 0.230 | 0.401 | 0.790 |
|  | *Fis* | 0.034 | 0.193 | 0.086 | 0.213 | 0.155 | 0.034 | 0.170 | 0.141 | 0.148 | 0.039 | -0.048 |
|  | *Ar* | 6.125 | 5.536 | 5.572 | 5.882 | 5.644 | 6.202 | 3.062 | 3.795 | 4.686 | 4.196 | 5.879 |
| AFND30 | Hex | 1.000 | 0.986 | 0.960 | 0.646 | 0.620 | 0.874 | 0.972 | 0.720 | 0.636 | 0.846 | 0.977 |
|  | Hdef | **0.000** | 0.016 | 0.043 | 0.818 | 0.382 | 0.127 | 0.039 | 0.290 | 0.447 | 0.155 | 0.023 |
|  | *Fis* | 0.120 | 0.378 | 0.143 | -0.041 | 0.093 | 0.058 | 0.237 | 0.000 | 0.011 | 0.150 | 0.049 |
|  | *Ar* | 7.238 | 6.523 | 7.213 | 7.549 | 7.435 | 7.621 | 4.965 | 4.152 | 6.883 | 5.468 | 7.215 |
| FUNO | Hex | 0.745 | 0.076 | 0.357 | 0.425 | 0.209 | 0.987 | 0.003 | 0.946 | 0.089 | 0.122 | 0.642 |
|  | Hdef | 0.255 | 1.000 | 0.730 | 0.847 | 0.809 | 0.013 | 0.997 | 0.058 | 0.970 | 0.937 | 0.359 |
|  | *Fis* | 0.037 | -0.174 | -0.057 | -0.133 | 0.038 | 0.068 | -0.458 | 0.028 | -0.140 | -0.163 | 0.030 |
|  | *Ar* | 5.223 | 5.639 | 5.258 | 4.987 | 4.457 | 4.816 | 3.470 | 3.859 | 6.519 | 3.558 | 5.450 |
| FUNQ | Hex | 1.000 | 0.999 | 0.173 | 0.996 | 0.920 | 0.999 | 0.990 | 0.574 | 0.530 | 0.559 | 0.865 |
|  | Hdef | **0.000** | 0.003 | 0.839 | 0.066 | 0.082 | 0.001 | 0.010 | 0.484 | 0.516 | 0.467 | 0.135 |
|  | *Fis* | 0.367 | 0.552 | -0.094 | 0.304 | 0.205 | 0.200 | 0.121 | -0.093 | -0.070 | -0.046 | 0.038 |
|  | *Ar* | 5.156 | 4.756 | 3.819 | 3.778 | 4.088 | 4.259 | 5.384 | 3.515 | 4.828 | 3.877 | 3.755 |
| AFND12 | Hex | 1.000 | 0.863 | 0.993 | 0.998 | 0.998 | 0.999 | 0.998 | 0.221 | 0.971 | 0.022 | 0.987 |
|  | Hdef | **0.000** | 0.143 | 0.007 | 0.005 | 0.002 | 0.001 | 0.003 | 0.867 | 0.038 | 0.988 | 0.013 |
|  | *Fis* | 0.307 | -0.014 | 0.167 | 0.563 | 0.180 | 0.178 | 0.050 | -0.096 | 0.026 | -0.186 | 0.002 |
|  | *Ar* | 6.552 | 5.762 | 6.767 | 5.000 | 6.516 | 6.696 | 5.278 | 3.020 | 6.098 | 4.209 | 6.703 |
| AFND7 | Hex | 0.369 | 0.870 | 0.986 | 0.723 | 0.990 | 0.977 | **0.000** | 0.331 | 0.615 | 0.006 | 0.184 |
|  | Hdef | 0.632 | 0.162 | 0.015 | 0.292 | 0.010 | 0.023 | 1.000 | 0.669 | 0.391 | 0.996 | 0.816 |
|  | *Fis* | -0.043 | -0.016 | 0.109 | 0.195 | 0.057 | 0.154 | -0.613 | -0.158 | -0.099 | -0.162 | -0.107 |
|  | *Ar* | 5.506 | 3.924 | 5.354 | 6.000 | 5.364 | 5.254 | 3.169 | 3.552 | 5.408 | 4.369 | 5.296 |
| AFUB11 | Hex | 1.000 | 0.945 | 0.885 | 0.686 | 0.888 | 1.000 | 0.590 | 0.003 | 0.975 | 0.790 | 0.996 |
|  | Hdef | **0.000** | 0.084 | 0.118 | 0.472 | 0.115 | 0.001 | 0.499 | 0.999 | 0.026 | 0.233 | 0.004 |
|  | *Fis* | 0.223 | 0.170 | 0.088 | 0.056 | 0.222 | 0.046 | -0.082 | -0.452 | 0.111 | -0.108 | 0.184 |
|  | *Ar* | 4.611 | 5.551 | 5.799 | 5.000 | 4.900 | 4.888 | 3.899 | 2.500 | 4.786 | 3.567 | 5.214 |
| AFUB6 | Hex | 0.995 | 0.958 | 0.263 | 0.810 | 0.985 | 0.622 | 0.855 | 0.146 | 0.718 | 0.011 | 0.095 |
|  | Hdef | 0.005 | 0.049 | 1.000 | 1.000 | 0.039 | 0.423 | 0.147 | 0.968 | 0.409 | 1.000 | 0.919 |
|  | *Fis* | 0.135 | 0.581 | -0.161 | -0.105 | 0.446 | 0.058 | 0.323 | -0.232 | 0.002 | -0.459 | -0.054 |
|  | *Ar* | 3.204 | 2.930 | 2.630 | 3.000 | 2.878 | 2.905 | 2.829 | 2.000 | 3.941 | 2.276 | 2.903 |
| AFND5 | Hex | 1.000 | 1.000 | 0.244 | 0.954 | 0.086 | 0.985 | **NA** | 0.663 | 0.494 | 0.863 | 0.994 |
|  | Hdef | **0.000** | 0.001 | 0.824 | 0.060 | 0.938 | 0.015 | **NA** | 0.645 | 0.673 | 0.432 | 0.006 |
|  | *Fis* | 0.248 | 0.625 | -0.074 | 0.186 | -0.088 | -0.076 | NA | -0.031 | 0.006 | -0.098 | -0.038 |
|  | *Ar* | 4.262 | 3.871 | 3.086 | 4.889 | 3.872 | 3.668 | 1.000 | 2.000 | 4.012 | 2.000 | 3.741 |
| AFUB10 | Hex | 0.998 | 0.779 | 1.000 | 0.995 | 1.000 | 1.000 | 0.967 | 0.996 | 0.754 | 0.901 | 0.996 |
|  | Hdef | 0.002 | 0.496 | 0.000 | 0.020 | **0.000** | **0.000** | 0.043 | 0.004 | 0.274 | 0.139 | 0.004 |
|  | *Fis* | 0.170 | 0.366 | 0.283 | 0.350 | 0.470 | 0.201 | 0.323 | 0.436 | 0.120 | 0.179 | 0.247 |
|  | *Ar* | 6.720 | 3.987 | 7.318 | 6.660 | 5.939 | 6.376 | 3.645 | 5.371 | 7.760 | 3.409 | 6.028 |
| AFUB12 | Hex | 0.999 | 0.968 | 0.997 | 0.717 | 1.000 | 0.987 | **NA** | 0.974 | 0.725 | 0.263 | 0.891 |
|  | Hdef | 0.001 | 0.068 | 0.003 | 0.342 | **0.000** | 0.014 | **NA** | 0.035 | 0.312 | 0.740 | 0.109 |
|  | *Fis* | 0.302 | 0.321 | 0.484 | 0.094 | 0.364 | 0.142 | NA | 0.306 | 0.035 | -0.342 | 0.074 |
|  | *Ar* | 3.371 | 2.972 | 4.468 | 3.889 | 3.648 | 2.947 | 1.000 | 2.440 | 2.987 | 2.859 | 3.135 |
| FUNF | Hex | 0.814 | 0.204 | 0.245 | 0.649 | 0.207 | 0.530 | 0.594 | 0.174 | 0.480 | 0.013 | 0.740 |
|  | Hdef | 0.188 | 0.947 | 0.784 | 0.739 | 0.804 | 0.474 | 0.578 | 0.854 | 0.599 | 0.989 | 0.261 |
|  | *Fis* | 0.007 | 0.018 | -0.031 | -0.018 | -0.029 | 0.012 | 0.055 | -0.099 | 0.023 | -0.336 | 0.089 |
|  | *Ar* | 4.358 | 5.597 | 5.009 | 4.987 | 4.176 | 4.402 | 2.615 | 4.308 | 3.839 | 2.999 | 3.682 |
| HWE P < 0.05 | | 9 | 5 | 6 | 4 | 7 | 10 | 5 | 6 | 2 | 5 | 8 |
| Bonferroni correction | | 7 | 2 | 5 | 2 | 6 | 8 | 4 | 4 | 0 | 3 | 4 |

Hex, excess of heterozygosity; Hdef, heterozygosity deficiency; Hardy Weinberg equilibrium (HWE) test not in equilibrium are shown in bold; Fis, inbreeding coefficient; Ar, allelic richness; tests with P < 0.05 and those that remained significant following Bonferroni correction are shown at the end of the table.
