# Supplementary figures and images for: Population genetics of Anopheles funestus, the African malaria vector, Kenya
Source: Parasit Vectors. 2019 Jan 8;12:15. doi: 10.1186/s13071-018-3252-3 (PMC6323828; doi:10.1186/s13071-018-3252-3)

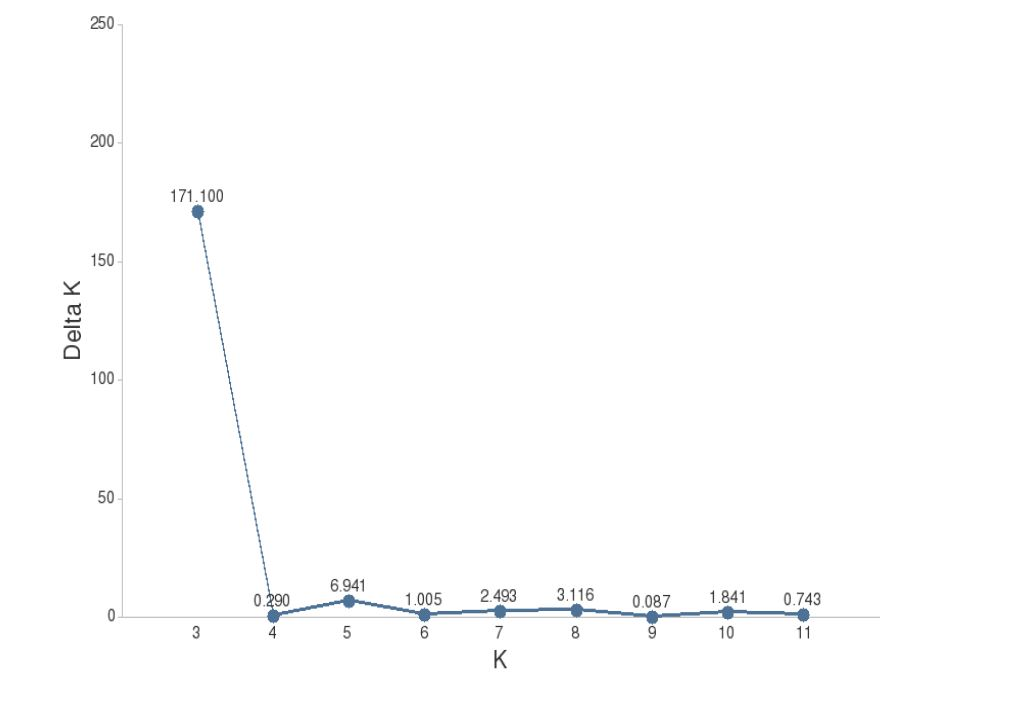

Supplement: Supplementary file 2 — Figure S1. Evanno delta K, STRUCTURE results for K = 3 based on microsatellite clustering analysis. (TIF 118 kb) [file 13071_2018_3252_MOESM2_ESM.tif]
